# Overweight, obesity, and risk of hospitalization for COVID-19: A community-based cohort study of adults in the United Kingdom

**DOI:** 10.1073/pnas.2011086117

**Published:** 2020-08-11

**Authors:** Mark Hamer, Catharine R. Gale, Mika Kivimäki, G. David Batty

**Affiliations:** ^a^Division of Surgery and Interventional Science, Faculty Medical Sciences, University College London, London WC1E 6BT, United Kingdom;; ^b^Medical Research Council Lifecourse Epidemiology Unit, University of Southampton, Southampton SO16 6YD, United Kingdom;; ^c^Lothian Birth Cohorts, Department of Psychology, University of Edinburgh, Edinburgh EH8 9JZ, United Kingdom;; ^d^Department of Epidemiology and Public Health, University College London, London WC1E 6BT, United Kingdom

**Keywords:** infection, obesity, COVID-19, epidemiology

## Abstract

The role of obesity and overweight in occurrence of COVID-19 is unknown. We conducted a large-scale general population study using data from a community-dwelling sample in England (*n* = 334,329; 56.4 ±8.1 y; 54.5% women) with prospective linkage to national registry on hospitalization for COVID-19. Body mass index (BMI, from measured height and weight) was used as an indicator of overall obesity, and waist−hip ratio for central obesity. Main outcome was cases of COVID-19 serious enough to warrant a hospital admission from 16 March 2020 to 26 April 2020. Around 0.2% (*n* = 640) of the sample were hospitalized for COVID-19. There was an upward linear trend in the likelihood of COVID-19 hospitalization with increasing BMI, that was evident in the overweight (odds ratio, 1.39; 95% CI 1.13 to 1.71; crude incidence 19.1 per 10,000) and obese stage I (1.70;1.34 to 2.16; 23.3 per 10,000) and stage II (3.38; 2.60 to 4.40; 42.7 per 10,000) compared to normal weight (12.5 per 10,000). This gradient was little affected after adjustment for a wide range of covariates; however, controlling for biomarkers, particularly high-density lipoprotein cholesterol and glycated hemoglobin, led to a greater degree of attenuation. A similar pattern of association emerged for waist−hip ratio. In summary, overall and central obesity are risk factors for COVID-19 hospital admission. Elevated risk was apparent even at modest weight gain. The mechanisms may involve impaired glucose and lipid metabolism.

Existing epidemiological data on obesity and infectious respiratory diseases are inconsistent. Various cohort studies have shown overweight and obesity to be associated with both increased (1, 2) and decreased risk (3) of community-acquired pneumonia and other upper respiratory tract infections, and also to protect against mortality from pneumonia (4–6). While a link between obesity and the occurrence of COVID-19 infection may be biologically plausible, to date, only prognostic studies based on small clinical samples have been conducted (7–9). Findings suggest that higher-weight patients experience increased rates of progression to intensive care (7, 8). It is unclear whether a potential obesity–COVID-19 association is driven by underlying morbidity or other biological mechanisms. Accordingly, we examined the aetiological relation of overweight and obesity with new cases of COVID-19 hospitalizations in a general population-based cohort study with available biomarker data.

## Methods

### Study Population.

Baseline data were collected in the UK Biobank study during 2006 to 2010 across 22 research assessment centers in the United Kingdom (*n* = 502,655; aged 40 y to 69 y; response rate 5.5%) (10, 11). Ethical approvals were received from the North-West Multicenter Research Ethics Committee, and participants provided informed consent. Data have been publicly deposited (https://www.ukbiobank.ac.uk/) and are available through an application process.

### Obesity Measures.

Body weight was measured using a Tanita BC418MA scale (12). Nurses measured standing height using a Seca height measure with the head positioned in Frankfort plane. Body mass index (BMI) was calculated using the usual formula (weight [kilograms]/height squared [meters squared]) and categorized into five standard groups: underweight BMI, <18.5 kg/m^2^; reference category, 18.5 kg/m^2^ to <25 kg/m^2^; overweight, 25 kg/m^2^ to <30 kg/m^2^; obese stage I, 30 kg/m^2^ to < 35 kg/m^2^; and obese stage II, ≥35kg/m^2^. Waist-to-hip circumference was measured with a Seca 200 measuring tape using standard procedures. A waist-to-hip ratio (WHR) of ≥0.9 in men and ≥0.8 in women was used to denote central obesity.

### Covariates.

During the clinic visit, data were collected via self-report for age, sex, ethnicity (White, South Asian, Black, Chinese, other), smoking history (never, previous, current), frequency of alcohol intake (daily or almost daily, one to two times a week, rarely, never/ex-drinker), educational attainment (college/degree educated; non-degree educated), types of physical activity in the last 4 wk (none, walking, exercise and sport, household maintenance work and gardening), and self-reported physician-diagnosed cardiovascular disease (CVD) and diabetes. Further clinical data included resting seated blood pressure and a fasting blood sample from which various analytes were assessed, including total cholesterol, high-density lipoprotein (HDL) cholesterol, glycated hemoglobin (HbA1C), and C-reactive protein (CRP) (13). Hypertension was defined as elevated blood pressure (≥140/90 mmHg) and/or use of antihypertensive medication.

### Linkage of Hospitalization Data for COVID-19.

The UK health care system, National Health Service, is funded from taxation to provide comprehensive health care coverage available to all legally registered UK residents. Data on COVID-19 status were obtained from Public Health England covering the period from 16 March 2020 up to 26 April 2020. During this period, testing was restricted to those with symptoms in hospital (https://biobank.ndph.ox.ac.uk/showcase/field.cgi?id=40100); thus, our outcome represents hospitalizations for severe COVID-19. The data cover England only; thus, participants residing in Scotland and Wales were removed from our analytical sample. Biological samples from combined nose/throat swabs were used to perform COVID-19 testing, with real-time PCR in accredited laboratories (14).

### Statistical Analyses.

Logistic regression was used to examine associations between BMI, central obesity, and COVID-19. We undertook separate analyses, firstly treating BMI or WHR as categorical variables and secondly as a continuous variable (per SD). Odds ratios (OR) were first adjusted for age and sex, followed by smoking, physical activity, alcohol, education, ethnicity, diabetes, hypertension, and CVD. A final adjustment to explore intermediate mechanisms included the biomarkers total cholesterol, HDL cholesterol, HbA1C, and CRP. Analyses were performed using SPSS Version 26.

## Results

The sample contained 334,329 participants (56.4 ±8.1 y; 54.5% women) who were alive prior to COVID-19 testing (5 March 2020), and had available data on BMI and covariates. Around 0.2% (*n* = 640) of the sample were hospitalized with a COVID-19 infection. Participants were largely (94.5%) White British, 66.6% were overweight or obese, 9.8% were smokers, 4.8% had a diabetes diagnosis, 56.1% had hypertension, and 5.1% had CVD (heart attack, angina, or stroke). In fully adjusted models, we observed independent associations between several covariates and COVID-19, including age, male sex, smoking, physical inactivity, non-White ethnicity, and alcohol (Table 1).

**Table 1.** Association of baseline obesity, covariates, and biomarkers with hospital admission for COVID-19 (*n* = 334,329)

		OR (95% CI)		
	N cases/N total	Model 1	Model 2	Model 3
Obesity classification (BMI categorical) (1)				
Underweight	2/1,650	1.06 (0.26, 4.29)	0.99 (0.24, 3.99)	1.07 (0.26, 4.33)
Normal	137/110,091	1.0 (Ref)	1.0 (Ref)	1.0 (Ref)
Overweight	273/142,889	1.39 (1.13, 1.71)	1.27 (1.03, 1.56)	1.18 (0.95, 1.47)
Obese I	134/57,691	1.70 (1.34, 2.16)	1.37 (1.06, 1.75)	1.20 (0.92, 1.56)
Obese II	94/22,008	3.38 (2.60, 4.40)	2.37 (1.78, 3.14)	1.95 (1.44, 2.65)
BMI continuous variable (2)				
Per SD (4.8 kg/m^2^) increase	640/334,329	1.38 (1.29, 1.47)	1.27 (1.18, 1.36)	1.21 (1.11, 1.31)
Covariates				
Age (per 5 y)	640/334,329	1.08 (1.03, 1.13)	1.08 (1.02, 1.13)	1.07 (1.01, 1.13)
Male sex	366/152,162	1.57 (1.34, 1.84)	1.60 (1.35, 1.88)	1.40 (1.16, 1.68)
Smoking	87/32,899		1.63 (1.27, 2.08)	1.54 (1.21, 1.98)
Physical (in)activity	78/19,690		1.52 (1.19, 1.95)	1.57 (1.23, 2.01)
Alcohol abstainer	77/25,091		1.61 (1.24, 2.09)	1.47 (1.18, 1.91)
Non-White ethnicity	69/16,964		2.06 (1.58, 2.70)	2.00 (1.53, 2.61)
Non-degree educated	473/225,134		1.17 (0.98, 1.40)	1.16 (0.97, 1.39)
Diabetes	57/16,101		1.07 (0.81, 1.42)	0.80 (0.58, 1.10)
CVD	64/17,164		1.34 (1.02, 1.76)	1.22 (0.92, 1.60)
Hypertension	416/188, 041		1.08 (0.90, 1.29)	1.09 (0.91, 1.30)
Biomarkers				
Total cholesterol per SD (44.1 mg/dL)	640/334,329			0.94 (0.86, 1.03)
HDL cholesterol per SD (14.7 mg/dL)	640/334,329			0.86 (0.77, 0.96)
HbA1C per SD (6.5 mmol/mol)	640/334,329			1.07 (1.03, 1.12)
Log-CRP per SD (doubling)	640/334,329			1.04 (0.96, 1.13)

Two separate analyses were undertaken: 1) treating BMI as a categorical variable and 2) BMI as a continuous variable (per SD). Model 1: Adjusted for age (per 5 y) and sex. Model 2: As Model 1, and additionally adjusted for smoking, physical activity, alcohol consumption, education, ethnicity, diabetes, hypertension, and CVD. Model 3: Additionally adjusted for total cholesterol, HDL cholesterol, glycated hemoglobin (HbA1C), and high-sensitivity CRP.

There was a linear increase in the risk of COVID-19 with increasing BMI, that became evident from modestly elevated weight (overweight category) to stage II obesity compared to normal weight (Table 1). Associations were little attenuated after adjustment for confounding factors or possible intermediate mechanisms such as comorbidity. We observed a similar pattern of results for central obesity assessed from WHR (OR = 1.43; 1.20, 1.71) after adjustment for covariates, that was linear when modeled as a continuous variable (fully adjusted per SD [0.1 units], OR = 1.29; 1.15, 1.44).

We performed further analyses to examine possible biological mechanisms. In linear regression models adjusted for covariates, BMI (per SD increase) was associated with HbA1C (B = 0.73; 0.71, 0.74) and HDL cholesterol (B = −0.11; −0.10, −0.12). These biomarkers were predictive of COVID-19 in a dose–response manner (Table 1). After additionally controlling the obesity–COVID-19 association for biomarkers (Table 1), these adjustments reduced the magnitude of the relationship by 33 to 46%; for example, the OR for stage II obesity and COVID-19 dropped from 2.37 (95% CI, 1.78, 3.14) to 1.95 (95% CI, 1.44, 2.65); the attenuation in effect estimates was largely driven through HbA1C and HDL (Table 1).

Given the reported increased risk of COVID-19 in ethnic minority groups, we restricted the analysis to White participants. The pattern of results remained the same: Increased risk of COVID-19 was observed across the overweight (OR 1.18; 95% CI, 0.98, 1.44), obese stage I (1.40;1.12, 1.76), and morbidly obese (1.90; 1.44, 2.50) compared to normal weight in adjusted models.

## Discussion

In this aetiological study, we found associations between obesity and higher odds of COVID-19 with severe symptoms requiring hospitalization in a large community-dwelling cohort that are consistent with the few prognostic studies of smaller clinical samples (7–9). The results were robust to adjustment for demographic characteristics including ethnicity and self-reported cardiometabolic diseases. However, adjustment for biomarkers, such as hemoglobin A1C and HDL cholesterol, attenuated the association by 33 to 46%, suggesting the mechanisms may involve impaired glucose and lipid metabolism. The accumulation of differentiated cytotoxic T cells have been linked to impaired glucose homeostasis in previous work (15), and we have also demonstrated associations between HbA1C and Cytomegalovirus infection (16). Thus, impaired glucose regulation appears to be a plausible mechanism, and the links between obesity and COVID-19 infection may be more complex than simple mechanical aspects of excess fat on diaphragm contractility.

A key strength is that measures of adiposity were collected at least 10 y before infection, thus ruling out possible reverse causation, that is, infection resulting in weight loss rather than the converse. This issue is of particular concern in prognostic studies of patient samples that may have already suffered significant weight loss from the illness prior to the point of admission. Weight change might have occurred during follow-up, causing misclassification. However, in a subsample (*n* = 19,772) with repeat assessment after a median of 4.4 y, BMI remained relatively stable (baseline, 26.9 ± 4.5 vs. follow-up, 27.0 ± 4.6 kg/m^2^; Pearson *r* = 0.93). We captured COVID-19 cases of sufficient severity to warrant in-patient care, although we did not undertake cohort-wide testing; thus, true prevalence remains unknown. By virtue of the fact that obese participants are likely to present with more risk factors, these patients may have been prioritized for testing. The low response rate (5.5%) to the original baseline survey in UK Biobank may have introduced bias, as participants were generally healthier and better educated than the general population. However, this is unlikely to influence risk factor−disease associations (17).

In conclusion, we observed a higher likelihood of COVID-19 hospitalization with increasing overall and central adiposity, even in participants with modest weight gain. Since over two-thirds of Westernized society are overweight or obese, this potentially presents a major risk factor for severe COVID-19 infection and may have implications for policy.

## Data Availability

Anonymized (individual participant characteristics) data have been deposited in UK Biobank under Application 10279 (https://www.ukbiobank.ac.uk/).
